# Situational and Dispositional Determinants of Intentional Deceiving

**DOI:** 10.1371/journal.pone.0019465

**Published:** 2011-04-29

**Authors:** Maria Serena Panasiti, Enea Francesco Pavone, Arcangelo Merla, Salvatore Maria Aglioti

**Affiliations:** 1 Psychology Department, Sapienza – University of Rome, Rome, Italy; 2 IRCCS Santa Lucia Foundation, Rome, Italy; 3 Department of Neuroscience and Imaging, G. d'Annunzio University, Chieti, Italy; 4 Institute for Advanced Biomedical Technologies, University G. d'Annunzio Foundation, Chieti, Italy; University of Maribor, Slovenia

## Abstract

Does opportunity make the thief or are people dispositionally prone to deceive? The interaction between personality and the circumstances surrounding deception is crucial to understand what promotes dishonesty in our society. Due to its inherent spontaneity and sociality, deceptive behaviour may be hardly reproducible in experimental settings. We developed a novel paradigm in the form of an interactive game where participants can choose whether to lie to another person in situations of loss vs. gain, and of no-reputation-risk vs. reputation-risk linked to the disclosure of their deceptive behaviour to others. Thus, our ecological paradigm allowed subjects to spontaneously decide when to lie and face the challenge of deceiving others. In the case of loss, participants lied to reverse the outcome in their favour. Deception was lower in the reputation-risk condition where personality traits concerning social interactions also played an important role.

The results suggest that deception is definitely promoted by unfavourable events, and that maintaining one's own reputation encourages honesty, particularly in socially inclined individuals.

## Introduction

As we think about political lies, tax evasion or swindlers, we realize that social interactions are very often permeated by deceptive behaviours. Although this behaviour is publicly condemned [1] people keep telling lies. The issue of the spread of dishonesty among societies has been addressed from several disciplines like psychology [2], [3], [4] and social psychology [5], [6], [7]. Importantly, the evolutionary game theory described the role of defection and cooperation in solving social dilemmas [8], [9], [10]. Yet, little is known about the circumstances under which this behaviour is promoted and how personality dispositions influence situational deception. Studies suggest that deciding whether to deceive involves a motivational conflict between the temptation to dishonestly achieve some benefit and the desire to act in a social appropriate manner [7]. Experimental research on deception has to face with the challenge of devising tasks that can induce such a conflict in a naturalistic way. The majority of the paradigms used thus far in the neuroscientific study of deception instruct subjects when to lie and do not provide a partner to lie to. Thus, these paradigms largely neglect two fundamental aspects of deception namely intentionality and sociality (for a review see [11]). More ecological paradigms in which subjects could spontaneously deceive [12], [13] other people [14] have been used only recently. Here we devised a novel experimental paradigm able to induce the conflict that people experience when faced with the choice to deceive or not another person and tested the effect on deceptive behaviour of both situational and dispositional variables. It is well known, for example, that deciding to be dishonest depends on the circumstances as the same person can decide to cheat during an academic exam but never evade taxes, or he/she could lie about his/her curriculum but never lie to his/her friends. Moreover, the circumstances where the people's goals could not be accomplished non-deceptively, promote lying behaviour [2].

It has also been demonstrated that people deceive less when the potential monetary reward related to cheating is significantly high thus making the circumstance morally challenging; or when cues to their moral standards are provided [6]. In contrast, people deceive more when the monetary reward for cheating is replaced by tokens and the moral implications of the circumstance can be reinterpreted [6].

Among the situations that may change the tendency to lie, reputation management plays an important role. People care so much about how they are regarded by others that acquiring a good reputation activates reward-related brain areas [15]. Moreover, building and maintaining one's own reputation plays an important role in promoting cooperation and prosocial behaviour [16], [17]. In a similar vein, the presence of potentially disapproving people or authority figures (i.e. of external cues to self-regulation) elicits a reduction in the expression of racial bias [18], [19], [20]. Importantly, under realistic deception situations people risk to loose their social capital [21]. Thus, the decision to deceive has to take into account the ability to manage one's own reputation. Despite the importance of this variable, no study has investigated the role of reputation management on deception thus far.

Another important determinant of deception is the dispositional tendency to behave according to one's own individual differences in personality traits. Studies indicate, for example, that people who self-reported to tell more lies during their everyday life scored high in impression management, manipulativeness and extroversion scales [3]. Likewise, manipulative people when asked about their lie-telling behaviour in everyday life, seem not to perceive themselves as frequent liars, and report to feel less guilty about their lies and comfortable when imagining to lie in high-stakes situations [22].

No systematic evidence for the comparative influence of situational and dispositional variables on deceptive behaviour has been provided. In the present study, we investigate how the decision to deceive another individual is influenced by two important situational variables (namely favourable vs. unfavourable reality and the reputation risk vs. no reputation risk) and by dispositional individual differences (personality traits). We created an experimental situation in which subjects were free to decide whether to tell the truth or to lie to an opponent player in order to obtain an economical benefit (self-gain lies) or to help another individual (altruistic lies). Moreover, we measured the impact of reputation on this decision. The participants' concern about their reputation was induced by telling them that the other player would be aware of their decision. Moreover, we tested whether the perceived reputation risk and the deceptive behaviour was enhanced in subjects who played with the opponent seated in the same room (Presence-Group) with respect to those who played with the opponent seated in a different room (No-Presence Group). Specifically, we asked whether facing the event of a loss would promote deception among participants (as measured by the number of self-gain lies) and whether the impact of reputation would decrease self-gain lies and increase altruistic lies.

To investigate the interactions between personality and deceptive behaviour, we examined extensively the participants' personality (by means of the short form of Temperamental and Character Inventory, [23]). Moreover, we examined manipulativeness, moral disengagement, impression management and self-deception as individual differences dimensions that we expected to be important predictors of deception in social interactions.

## Methods

### Participants and Design

Fifty-two participants (26 females, age between 19 and 29 years, mean = 24.38) were recruited. Two participants were excluded from the analysis because they did not believe they were playing against a real person. Twenty-six of them (13 females) played in the *No-Presence Group* and twenty-four (12 females) played in the *Presence Group*. Subjects were paid 10 euros for their participation and had the possibility to extra win up to 30 euros during the game. All the subjects signed written informed consent prior to be enrolled. The experimental protocol was approved by the independent Ethics Committee of the Santa Lucia Foundation (Scientific Institute for Research Hospitalization and Health Care) and was carried out in accordance with the principles of the 1964 Declaration of Helsinki.

### Experimental Task

Subjects (Ss) performed in a two-cards game where an ace of heart was associated with gain, and an ace of spades was associated with loss. Each subject was instructed that the opponent player (OP) was the first mover and was supposed to choose one of the two covered decks without knowing the outcome of the choice, which would be communicated by the S. By lying, S had the chance to reverse the outcome and thus to win when he/she had actually lost (*self-gain lie*) or to lose when he/she had actually won (*altruistic lie*). Trials in which the S was supposed to gain or to lose were defined as Favourable and Unfavourable Reality respectively. S performed the game in two conditions: the Reputation-Risk (R), in which S knew that OP was informed about his/her lies; and the No-Reputation Risk (NR) in which S knew that the OP was not informed. The order of the two conditions was counterbalanced across participants. The two players had 25 euros each for playing. For each trial, the winner took money from the other player's payoff. S were told that the amount of gain/loss on each trial was arbitrarily decided by a computer algorithm. The precise amount of gain/loss was communicated only at the end of the task. This allowed to rule out that the subjects' behaviour was based on a trial by trial computation of gain/loss.

### Materials and Procedure

**Figure 1:**
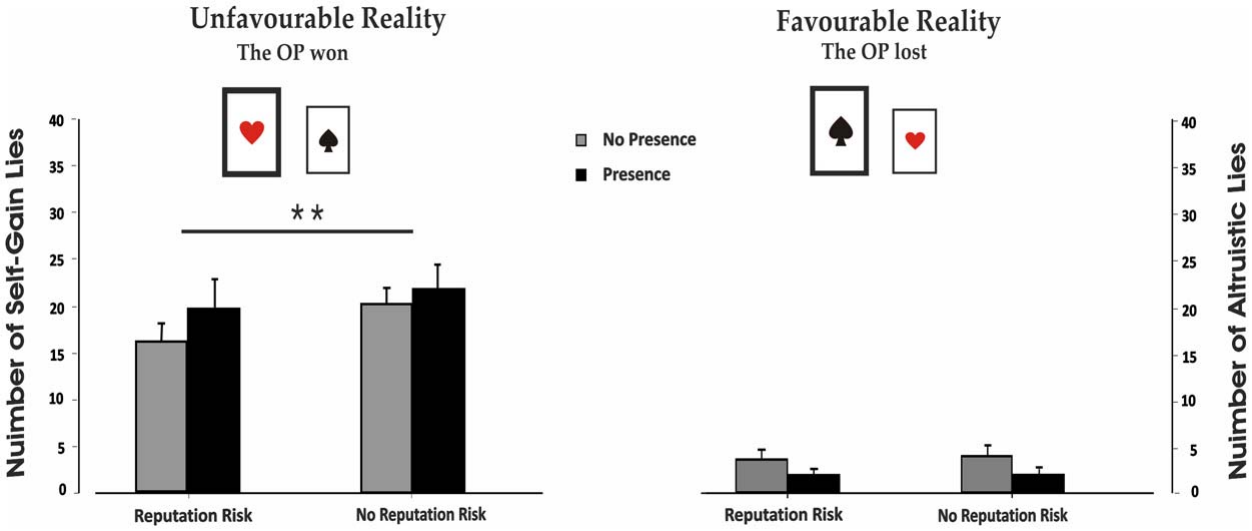
Number of lies. Self-gain and altruistic lies (mean ± standard error) produced by the two subject groups (No-Presence Group, *grey bars;* Presence Group, *black bars*) in the two possible opponent (OP) choice outcomes (*favourable/unfavourable*) in the two conditions (*Reputation Risk/No-Reputation Risk*) are reported. The number of Self-Gain lies is significantly reduced in the Reputation Risk Condition (p = .01).

Subjects were seated comfortably in an acoustically shielded room. Stimulus presentation timing and randomization were controlled by using E-prime ver.1.2 software (Psychology Software Tools Inc., Pittsburgh, PA) running on a PC. Participants sat 57 cm away from a 22 inch LCD monitor where the stimuli, two play cards consisting in an ace of heart and an ace of spades, appeared on a white background (see figure 1). Each trial started with the presentation of a central fixation cross lasting 1000 msec, followed by the presentation of the stimuli. The left/right position of the heart/spades ace was counterbalanced. After a varying time interval (between 2000 and 3000 msec), one of the two cards became bigger, indicating the OP's choice. This randomized interval was employed for the No-Presence Group to provide tangible probes that the OP was a real person. For the Presence-Group, the actor triggered the stimulus presentation by clicking a mouse. After each OP's choice, participants were asked to press on a keypad either the “V” key to communicate the truth (the italian word for truth is verità) or the “M” key to lie (the italian word for lie is menzogna). The stimulus remained visible on the screen until the response was given. Each block contained 80 trials, half of them providing the Unfavourable Reality (the OP won), the other half the Favourable Reality (the OP lost).

To avoid any spurious influence, in no condition was the experimenter in the testing room.

### No-Presence of the OP (Group 1)

Subjects were told that they were going to play an online card game with another participant situated in a different room of the building and that they would meet the other player at the end of the game.

### Physical Presence of the OP (Group 2)

After reading the task instructions, subjects were blindfolded while the other participant (the actor) entered the room. Once subjects were seated, they could see only the computer screen in front of them and had no chance to look at the other player. This procedure assured us that the actor's physical features would not influence the subjects' performance. In addition, we informed Ss that they were not allowed to talk and that thanks to a microphone in the room the examiner could stop the experiment in the event that someone spoke. That S did not actually talk was double checked by asking the actors in the room. Importantly, unbeknownst to the subjects, the OP choice was controlled by a computer.

### Manipulation Check

After the game, participants were qualitatively debriefed about the interaction. We asked them questions like: “Did you enjoy the game?”; “Has your opponent been lucky?”; “Was he/she luckier in the first or in the second block?”. The two subjects who declared they did not believe or were sceptical about the fact that the OP was a real player were excluded from the analysis.

### Personality Measures

After the manipulation check, subjects performed the electronic version of the short form of Temperamental and Character Inventory designed to assess four temperamental (*Novelty Seeking; Harm Avoidance; Reward Dependence; Persistence*) and three character (*Self-Directedness; Cooperativeness; Self-Transcendence*) dimensions [23]. Moreover, subjects were administered the Balanced Inventory of Desirable Responding (BIDR) [24], the Machiavellanism Scale (MACH IV) [25] and the Moral and Civic Disengagement [26].

## Results

### Binomial Analysis (Truth vs. Lie)

The number of lie and truth responses was collected and used as dependent variable. The percentage of Truth responses in the NR block for both groups (No-Presence: 70%; Presence: 70%) was higher than the percentage of Lie responses (binomial test, No-Presence: Truth/Lie, p<.01; Presence: Truth/Lie, p<.01). Also in the Reputation-Risk Block the percentage of Truth responses was higher for both groups (No Presence: 75%; Presence: 73%; binomial test, No Presence: Truth/Lie, p = .01; Presence: Truth/Lie, p = .01) (**Table 1** **, first and second row**).

**Table 1:** Percentage of Truth and Lie responses in each condition.

	NO-PRESENCE GROUP			PRESENCE GROUP		
	Lie	Truth	p	Lie	Truth	p
**REPUTATION RISK**	25%	75%	= .01	27%	73%	= .01
**NO REPUTATION RISK**	30%	70%	<.01	30%	70%	<.01

		Lie	Truth	p	Lie	Truth	p
**FAVOURABLE REALITY**		10%	90%	<.01	5%	95%	<.01
**UNFAVOURABLE REALITY **		47%	53%	** = .12**	52%	48%	** = .12**

The null hypothesis in the binomial test is the case in which two categories are equally likely to occur. When this test is statistically significant one category is much likely to occur than the other. Our data show that the truth responses are significantly more likely to occur in all conditions except in Unfavourable Reality i.e. when OPs won and Ss lost. In this case, lie and truth responses were comparable both in the No-Presence Group (p = .12) and in the Presence Group (p = .12).

Higher percentage of Truth than Lie responses was found for both groups in Favourable Reality condition (No-Presence 90%, Presence 95%; binomial test, No-Presence: Truth/Lie, p<.01; Presence: Truth/Lie, p<.01). By contrast, the percentage of truth and lie responses did not differ across groups in the Unfavourable Reality (binomial test, No-Presence: Truth/Lie, p = .12; Presence: Truth/Lie, p = .12) (**Table 1** **, third and fourth row**).

### Analysis of Deceptive Responses

A 2×2×2 analysis of variance (ANOVA) for mixed models with Group (No-Presence vs. Presence) as a between-subjects factor and Reality (Favourable vs. Unfavourable) and Reputation (Reputation Risk vs. No-Reputation Risk) as within-subject factors was run. ANOVA did not show any significance of Group F(1,48)  = .03, p = .87 nor its interaction with Reputation F(1,48)  = 1.22, p = .28, or Reality F(1,48) = 2.07, p = .16. Interestingly, a main effect of Reputation F(1,48)  = 10.93, p<.01, η^2^ = .19 was found revealing that subjects told more lies in the NR than in the R block. In addition, the main effect of Reality was significant F(1,48)  = 100.53, p<.001, η^2^ = .68. This effect is accounted for by the fact that subjects lied more in the Unfavourable Reality than in the Favourable Reality, thus producing more self-gain lies then altruistic lies. Crucially, a significant Risk × Reality interaction was found F (1,48)  = 7.58, p = .01, η^2^ = .14. The Newman-Keuls post-hoc test for multiple comparisons showed that whereas no difference in the amount of altruistic lies between the R block and the NR block was found, subjects produced more self-gain lies in the NR block respect to the R block (**Figure 1**).

### Personality Measures

**Figure 2:**
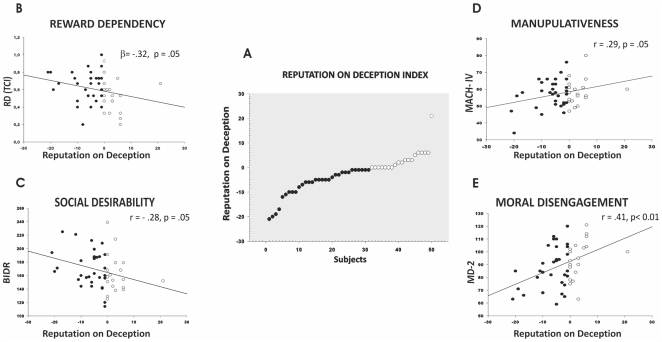
Correlations between personality traits and the impact of reputation on deception (RoD). A) the panel shows the RoD index for each subject. *Black dots* indicate subjects in whom deceptive behaviour was influenced by reputation risk. *White dots* indicate subjects in whom deceptive behaviour was not affected by reputation risk. The *left part* of the figure shows the negative correlations. B) indicates the TCI reward dependence scale is an independent predictor of RoD index. C) shows the significant negative correlation between RoD and Social Desirability Responding [Impression Management + Self-deceptive enhancement (BIDR)]. The *right part* of the panel show the positive correlations between RoD and Manipulativeness (MACH IV) (*D*) and Moral Disengagement (MD 2) (E). The higher impact of reputation (lower RoD), the higher the reward dependence and social desirable traits; the lower impact (higher RoD), the higher manipulativeness and moral disengagement traits.

Since the tendency to deceive was comparable in the two experimental groups, correlational analyses between self-gain or altruistic lies and personality traits were performed in all the subjects. No significant correlation was found. We sought to determine whether dispositional personality factors predicted how much reputation influenced deception. To this aim, we created a quantitative index of deception by subtracting Altruistic Lies from the Self-Gain Lies for each block. Then, we computed the impact of reputation on deception (RoD) as follows:
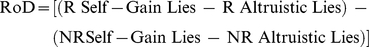



Negative and positive scores indicate more lies in the NR and the R blocks respectively (**Figure 2** **, A**).

The seven scales of TCI were entered in a standard multiple regression model as predictors, with the RoD as dependent variable. The regression model was significant (R = .52, adjR2 = .15, F (7,42)  = 2.22, p = .05). “Reward Dependence” (*ß* =  −.32, *t*42 =  −2.03, p = .05) was the independent predictor of the impact of reputation on deceiving (**Figure 2** **, B**). For regression analyses, we computed the Cohen's *f ^2^*: *R2*/(1 – *R2*), as an index of effect size. Cohen's *f ^2^* was computed on the adjusted *R2* (*f^2^* = .*18)*.

In addition, we found a significant negative correlation between RoD and the BIDR Total Score (r = −.28, p = .05) indicating that the higher the impact of reputation, the more the subjects responded according to social desirability (**Figure 2** **, C**). Furthermore, RoD correlated positively with MD2 (r = .41, p<.01) and MACH IV (r = .29, p = .05). Thus, subjects who showed high scores in moral disengagement (**Figure 2** **, D**) and manipulativeness (**Figure 2** **, E**) were less influenced by reputation risk while being deceptive.

## Discussion

We devised a novel experimental paradigm in the form of a cards game to investigate the effect of situations and dispositions in promoting deceptive behaviour in an ecological context where participants decided when to lie to another individual. Important situational variables were manipulated. Indeed, participants were faced with the moral dilemma of reverting their loss by lying to an opponent person who might or might not be in the same room. Moreover, the subjects' deceptive behaviour could or could not be disclosed to others and participants were thus informed that their reputation could or could not be at risk. Moreover, personality measures allowed us to assess the participants' disposition both in general and towards deception.

At least two key results concerning the situational manipulations were obtained. First, although participants were overall more truthful (more than 70% of total responses) than liar (around 30% of the total responses) and thus tended not to deceive others, a clear effect of Unfavourable Reality, i.e. of the situation where participants were more tempted to deceive, was found. In this latter situation, participants produced an equal number of lie and truth responses. Thus, the event of loss did increase deceptive behaviour although not to the point of inducing more lies than truth responses. That our participants in general did not lie as default is in keeping with an extensive study reporting that only a minority of 791 subjects tended to cheat and suggesting that the range of acceptable dishonesty was limited by the internal reward of being honest [6]. Importantly, although our paradigm did not abolish the participants' tendency to act in a socially appropriate and sensible manner, it was effective in pushing participants towards deception.

The second main result is that the reputation risk situation influenced the production of self-gain but not of altruistic lies. Studies indicate that altruistic lies, i.e. those produced to provide someone else's benefit at one's own cost, are rated as the most acceptable and likely to occur. In contrast, the self-gain lies, i.e. those involving the liar's benefit at others' cost, are rated as the least acceptable [4]. Different circumstances favour the production of one or the other type of lie. Guilty evoked by positive iniquity (i.e. when winning a lottery at the cost of others), for example, make people deceptively help others. In contrast, envy evoked by negative iniquity (i.e. when loosing a lottery to others' gain) leads people to deceptively hurt others [5].

According to the Theory of Self-Concept Maintenance, producing lies co-exist with the need to regard themselves as honest individuals [6]. Two main strategies permit to act dishonestly without affecting the positive concept of self. The first has to do with the subjects' tendency to categorize their own behaviour in a malleable way and thus to reinterpret reality in a self-serving manner finding a rationalization for dishonest actions. A clear reduction of deceptive behaviour is obtained by making very high the monetary gain that can be obtained by cheating. Indeed, in the above circumstances, the categorization of ones' own behaviour is less malleable and finding rationalizations for deception is much harder [6]. The second strategy is the inattention to one's own moral standards, implemented so as to avoid any link between self and dishonest actions [6]. In this vein, inducing subjects to pay attention to their own moral standards by asking them to recall the Ten Commandments or to read a code of honour, decreases the subjects' tendency to cheat [6].

In our experimental paradigm, subjects were told the interaction was just a game no matter whether reputation was or was not at risk. Since categorization malleability is comparable in two conditions, finding rationalizations for being deceptive might have been equally at play. Also, we reasoned that if reputation enhances attention to people's moral standard, deception might be classified as wrong no matter if produced with the intent to obtain self-gain or to help others. We found that reputation-risk decreased self-gain but did not increase altruistic lies. This suggests that, at least in our experimental conditions, is more important for people to appear honest than altruistic if this implies deception.

Indirect reciprocity, an important mechanism for the evolution of cooperation [27], consists in acts where the return for a favour comes from someone other than the recipient of the benefaction (i.e. “I scratch your back and someone else will scratch mine”). Helping others even when they cannot directly reciprocate, pays off anyway. Indeed, studies demonstrate that people who help another individual are more likely to receive help from others [16], [17]. It is relevant that indirect reciprocity may be linked to one's own reputation building [27]. Our subjects knew that the other player would have not be able to reciprocate directly their help. However, they could have spread out the voice about their honesty. Thus, we posit that a kind of indirect reciprocity might have played a role in the reputation condition of our study.

It is also worth noting that the pattern of deceptive behaviour was very similar when the opponent was or was not physically in the participants' same room during the interactive game. This is somewhat counterintuitive because the reputation risk should be higher when the opponent is in the same room. Indeed, reputation management can be easily induced in subjects even by subtle observation cues (i.e. pictures of eyes or eye-like stimuli) [28], [29]. A possible explanation for our result is that not allowing participants to have any physical contact with the other player made the same-room situation very similar to the online interaction. Therefore, it is likely that being aware that other people would know about the subject's behaviour has been sufficient to induce a strong concern about the deception-related reputation risk independently of whether the target of the deceptive behaviour was in the same or in a different room.

An interesting link between deceptive behaviour and dispositional tendencies was also found in our study. Indeed, the impact of reputation on self-gain deceptive behaviour turned out to be associated to a specific personality profile. In particular the participants who lied less in the reputation-risk situation were also highly reward dependent, i.e. they were very sensitive to signals of social approval [30]. Additionally, these subjects showed high social desirable responding (BIDR) traits, a measure that included both the intentional tendency of distorting one's own self by imaging to be perceived more favourably by others (Impression Management) and the unintentional tendency to portray oneself in a positive light (Self-Deception). This result is in line with studies showing that external cues to self-regulation influence the expression of prejudice selectively in people with specific personality traits [20], [31]. It has been shown, for example, that during a stereotype inhibition task performed in a private or in a public condition (where the experimenter was checking for signs of prejudice), a larger error-related positive component was elicited only in subjects who cared about their social image and tried to appear non-prejudiced [32]. Our data shows that also in the case of deception, the reputation has an impact only for people highly concerned about their social image. In addition, we found that the impact of reputation was less important for high manipulative and moral disengaged subjects. Manipulative people admit they cheat to get what they want [25] and moral disengaged people are particularly able to find moral self-sanction to their misbehaviours [33]. Taken together, our results have important practical implications. Indeed, in modern societies often permeated by deception, people need to understand on what circumstances one can promote or inhibit this kind of behaviour. We found that when people face unfavourable situations, the temptation to deceive becomes stronger. This tendency was not influenced by dispositional variables. Importantly, however, we found a clear link between personality dimensions and the reputation risk connected to the public disclosure of the deceptive behaviour. Mere knowledge of this risk seemed to work as an important restraint on deception. It must be noticed that in our paradigm subjects did not risk to be punished for their behaviour. Tellingly, evolutionary game theory studies [34], [35], [36] reviewed the role of reward and punishment in promoting public cooperation and found that under certain circumstances, reward and not only punishment can promote cooperation. This suggests that promoting a policy of transparency where the entire decision making process is carried out publicly and where building one's own reputation works as a social reward, could be a good deterrent for deception in social contexts. It is worth noting, however, that not all the people are affected by reputation. Thus, further investigation is needed to understand the complex interaction between dispositional and situational determinants of deception. As a final remark, we want to emphasize that the novel paradigm used in the present study turned out to be adept to induce a conflict between the temptation to deceive and the desire to act in a socially appropriate manner. Therefore, we propose it as a potentially very useful for testing lie and deception in ecological contexts at behavioural and neural levels.
